# Latitudinal changes in the lipid content and fatty acid profiles of juvenile female red squat lobsters (*Pleuroncodes monodon*) in breeding areas of the Humboldt Current System

**DOI:** 10.1371/journal.pone.0253314

**Published:** 2021-06-22

**Authors:** Fabián Guzmán-Rivas, Marco Quispe-Machaca, Dante Queirolo, Mauricio Ahumada, Ángel Urzúa

**Affiliations:** 1 Centro de Investigación en Biodiversidad y Ambientes Sustentables (CIBAS), Universidad Católica de la Santísima Concepción, Concepción, Chile; 2 Facultad de Ciencias, Departamento de Ecología, Universidad Católica de la Santísima Concepción, Concepción, Chile; 3 Facultad de Ciencias, Programa de Doctorado en Ciencias con mención en Biodiversidad y Biorecursos, Universidad Católica de la Santísima Concepción, Concepción, Chile; 4 Laboratorio de Tecnología Pesquera, Escuela de Ciencias del Mar, Pontificia Universidad Católica de Valparaíso, Valparaíso, Chile; Universidad de Antioquia, COLOMBIA

## Abstract

The red squat lobster *Pleuroncodes monodon* is a species of high commercial value that inhabits the Humboldt Current System. Along the Chilean coast, two populations are exploited by the fishing industry, one located off the coast of Coquimbo and the other off the coast of Concepción. Yet, it is unknown whether there are differences in the “bioenergetic fuel” (measured as lipid content and fatty acid profile) of juvenile populations of these two fishing units and whether these bioenergetic compounds can be modulated by differences in the environmental parameters (such as temperature or chlorophyll-a) of their breeding areas. To shed some light on this, we measured the lipid content and fatty acid profiles of the viscera and muscle of juvenile female red squat lobsters from these two fishing units, specifically from breeding areas near long-exploited fishing grounds: a) the northern fishing unit (NFU, from 26°S to 30°S) and b) the southern fishing unit (SFU, from 32°S to 37°S). We found differences in the lipid content, fatty acid profiles, and ratios of saturated fatty acids (C16:0/C18:0) of juvenile females from these two locations. In addition, the essential fatty acids (DHA/EPA) found in the viscera versus the muscle of these lobsters varied significantly. Juvenile females from the SFU (i.e. Concepción) showed a higher lipid content compared to the juvenile females from the NFU (i.e. Coquimbo). Consistently, individuals from the SFU had a higher content of fatty acids, which also proved to be richer in saturated and monounsaturated fatty acids compared to those from the NFU. Our results are important for the fisheries in both areas because these juvenile populations are the source of new recruits for the adult populations that are exploited by the fishing industry. Our study also aids in determining which populations are healthier or of better quality in bioenergetic terms. Furthermore, increasing the incorporation of bioenergetic parameters in fishery models is essential for the recruitment and stock assessment within an ecosystem approach, since it allows for the evaluation of the nutritional condition of different fishing populations.

## Introduction

In marine invertebrate species with a wide geographic distribution range, the quantification of morphometric parameters and bioenergetic reserves of individuals from different locations is necessary to assess the state of the species’ populations, which may differ among spatial scales [1–3]. These variations can be modified mainly by a) environmental parameters (e.g., temperature, food availability, salinity, dissolved oxygen, rainfall, etc.), b) geographic features (e.g., geomorphology, topography, and coastal dynamics), and c) ecological interactions (e.g., competition and depredation). As a consequence of these spatial differences, geographically separated populations can develop local adaptations that exhibit differences in their life history traits [more on this concept in: [4–12]]. On the other hand, marine invertebrate species that have a high dispersal potential (i.e., long-lived planktonic larvae) also have high levels of phenotypic plasticity [13]; they are able to adjust their morphology, physiology, and/or development in response to changes in their environment [14,15].

Some species of marine invertebrates in the Humboldt Current System (HCS) of the Southeast Pacific Ocean exemplify such spatial structure. The HCS is a highly productive ocean-coastal current system that ranges from 4°S (Ecuador) to 42°S (Chile) [16,17] and supports one of the largest fisheries worldwide [16]. Throughout the HCS there are several upwelling zones that differ from one another in intensity and duration, both of which can have an impact on the distribution, abundance, and characteristics of marine organisms [18–20]. Along the Chilean coast, the differences in the upwelling periods of the HCS have been reported in geographically separated and contrasting areas (e.g., the north, off the coast of Coquimbo vs. the south, off the coast of Concepción; [17–19]. In some cases, these differences have provoked adaptive plastic responses or even genetic breaks among individuals of the same species [21,22]. In this regard, populations of the same species inhabiting geographically distant regions may also exhibit differences in their life history traits due to the selective pressures of environmental parameters (such as temperature or food availability) [23]. These differences can mainly be observed morphologically [24] or in the fertility [3], biochemical composition [2], and biochemistry [25–27] of marine organisms.

An organism’s energy can be stored biochemically, primarily in the form of lipids and fatty acids [28,29]. These bioenergetic compounds play a key role in the nutrition, reproduction, and growth of many marine organisms [29–31]; and their availability can vary spatially, even within a single species due to biotic (e.g., nutrition, stage of development, competition, and/or food availability) and abiotic factors (e.g., temperature, upwelling regimes, and/or biogeochemistry at the continental shelf/habitat) that act upon individuals and populations [5,23,32–34]. Marine organisms with a wide biogeographic distribution that inhabit heterogeneous environments can show variations in the composition and profiles of fatty acids with latitude [35,36]. This variability in fatty acid profiles can result, for example, from variations in the essential fatty acids that are obtained through the organism’s diet and cannot be synthesized by the organism itself [31,37,38]. For instance, eicosapentaenoic (EPA, 20: 5ω3) and docosahexaenoic (DHA, 22: 6ω3) acids are highly unsaturated fatty acids, which are essential for organisms living at high latitudes (i.e., at low temperatures) because these fatty acids maintain fluidity and the optimal functioning of membranes [31,39]. In this regard, spatial variations in environmental parameters, such as temperature or food availability, can cause differences in the amount and concentration of several fatty acids [27,35].

We herein studied the red squat lobster (*Pleuroncodes monodon*), a key species in the marine food web [40] of the HCS and an important fishery resource [41]. *P*. *monodon* is widely distributed, from the Isla Lobos de Afuera (in Peru, ~7°S) to south-central Chile (~37°S) [42,43]. Additionally, its current biogeography is split within the Chilean HCS because it is almost nonexistent from 30°S to 32°S [44–46]. Hence, there are two geographically separated extraction units (or fishing grounds) where fishing vessels operate: one in the northern Chilean HCS (from 26°S to 30°S; i.e., the northern fishing unit “NFU”) and another in the southern Chilean HCS (from 32°S to 37°S; i.e., the southern fishing unit “SFU”) [40]. Within these fishing units, the adult populations of the NFU (Coquimbo) and those of the SFU (Concepción) have been intensively exploited by commercial fisheries in Chile [44,47,48]. The subsistence of these adult red squat lobster populations in Chile has only been achieved through the establishment of biological rest periods (during the months of January and February) and breeding areas in close vicinity to these historical fishing grounds (off the coast of Coquimbo and Concepción) [40,43,49]. These breeding areas are therefore considered to be the main sources of recruits for the adult populations, maintaining the fishing stock within these two large fishing units (for details on the concept of “sink”/“source” populations see: [50,51]). Besides, adult individuals should reflect similar biochemical conditions as the juvenile populations they came from, since the condition of early developmental stages (i.e., larval stages) can have long-lasting effects throughout an individual’s life-cycle (i.e., as juveniles and adults) [52,53].

The red squat lobster has a biphasic lifecycle, consisting of a pelagic larval phase and a benthic juvenile and adult phase [26,54]. It has five larval stages with a high dispersion capacity [15,55]. It reproduces throughout the year, except in the month of January; it also presents between three and four spawning periods during its annual cycle [54,56]. It therefore uses different energy sources throughout its development [40]. While the earliest larval stages feed on phyto- and zooplankton, benthic juveniles and adults have various feeding strategies: from filter-feeders to active herbivores, carnivores, scavengers, cannibals, and detritivores [40]. Moreover, food provides the bioenergetic fuel for individuals of any marine invertebrate and a surplus is mainly stored as lipids and fatty acids in the hepatopancreas and, to a lesser extent, in the muscle tissue [57]. However, the intake of food varies with habitat and prey [58], generally causing spatial differences in the biochemical composition of individuals related to the abundance of certain fatty acids, especially among species with wide distribution ranges [36].

In *P*. *monodon*, seasonal changes in environmental conditions are known to not only affect life history traits [56], but also the biochemical composition of adult specimens [26,27,48]. For instance, in cooler periods, adult individuals have higher biomass than during warmer periods [56]. Along these lines, studies on the seasonal variations of the nutritional condition of eggs [26,27], early larval stages [53,59], and adult individuals [26,27] have been conducted. However, the nutritional condition of juveniles within these two highly important breeding areas in Chile remains unknown. Nonetheless, knowledge on growth rates and size/body masses of populations of benthic resources is required for the maintenance of sustainable fisheries [60–62]. On the other hand, interest in the physiology and biochemistry of the larval and adult stages of *P*. *monodon* has substantially increased in recent years [26,27,48,53,56,59]. Nevertheless, relevant aspects regarding morphometric and bioenergetics parameters of the juvenile stages (onset of benthic phase) of this species, which are crucial for models on juvenile recruitment and fisheries’ management of adults, still remain unknown. Together with data on the nutritional condition of a species, improved management of fishery resources can be achieved through the development of sustainable exploitation strategies with an ecosystem approach, focusing, for instance, on populations with larger body sizes and better nutritional conditions within a widely distributed species [48,56,60,61,63]. Hence, the main objective of our study was to determine latitudinal variations in the lipid content and fatty acid profiles of juvenile females of the red squat lobster (*P*. *monodon*) from the two breeding areas in the Chilean HCS, located in the vicinity of its most exploited fishing grounds (the NFU at 29°S, and the SFU at 36°S). We hypothesized that the contrasting features of the two breeding areas of *P*. *monodon* would be reflected in variations of the morphometric parameters and bioenergetic reserves of its juvenile females. Hence, we expected that juvenile females from the SFU would have a greater body mass as well as a higher lipid and total fatty acid content than those from the NFU, due to the differences in key environmental parameters (such as temperature and chlorophyll-a) of these two sites.

## Materials and methods

### Description of the sampling area and collection of samples

**Fig 1:**
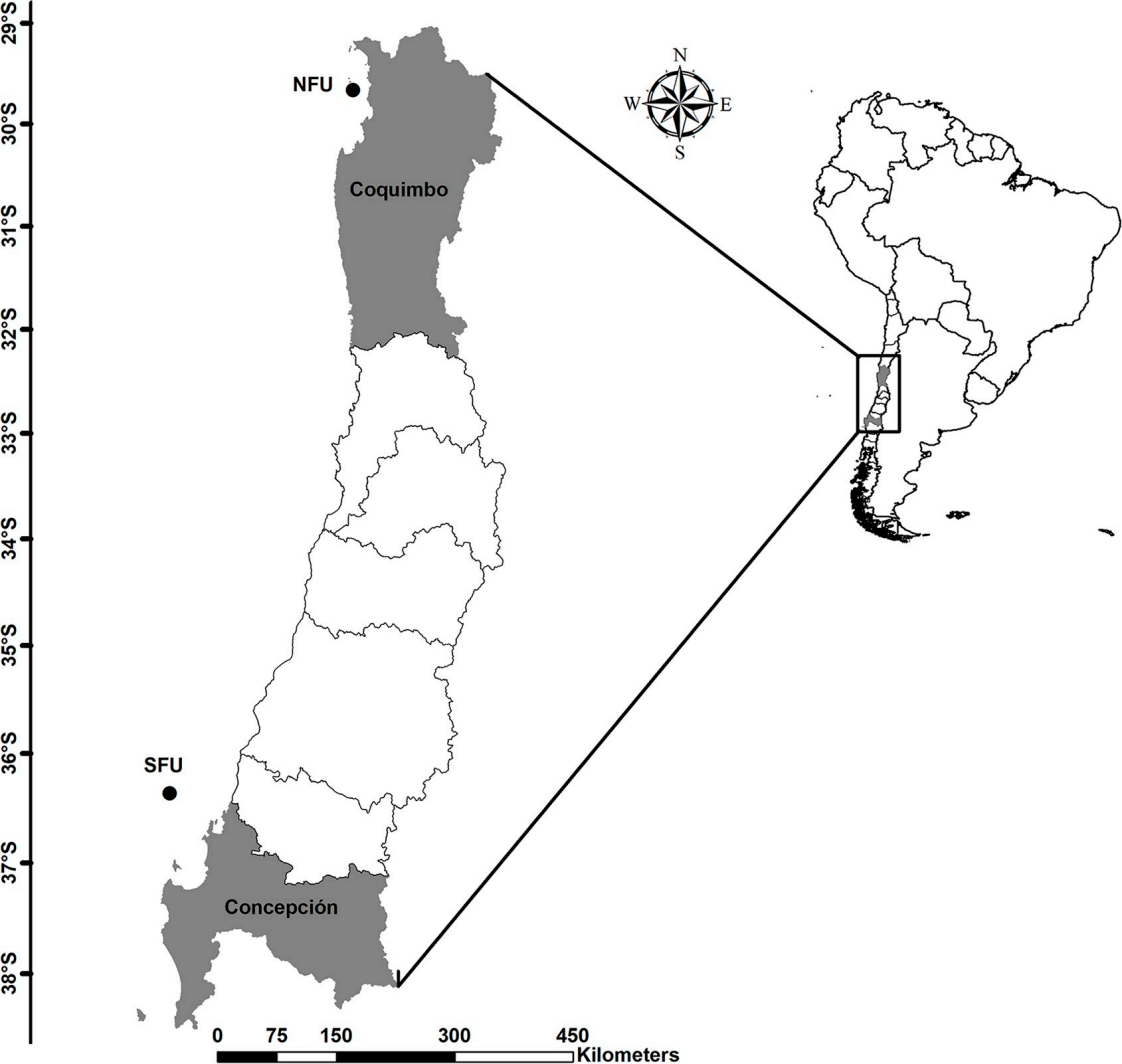
Sampling areas of the juvenile *Pleuroncodes monodon* female off the coasts Coquimbo and Concepción. The black dots correspond to the sampling areas. NFU, northern fishing unit; SFU, southern fishing unit.

Juvenile red squat lobsters were captured at depths of 80–100 m off the coast of Coquimbo (29° 58’ S; 71° 38’ W) and off the coast of Concepción (36° 22’ S; 73° 35’ W) during the month of May 2016 (Fig 1, we used ArcMap v10 software to create a map of the two sampling areas). Average sea surface temperature (SST) and Chl-a data (as proxies for the environmental conditions) of the study sites (i.e. NFU and SFU) for the year 2016 were obtained from the GIOVANNI website [64], which is administered by the National Aeronautics and Space Administration (NASA). In this study, Chl-a was used as a proxy for the availability of planktonic food [65], which is the main food source of juveniles, since they are filter feeders [40,49]. This data were used to compare the environmental parameters, the morphometric parameters (i.e. size and dry weight), the lipid content and the fatty acid profiles of juvenile females of the red squat lobster from the two studied breeding areas. The samples were part of a monitoring program for the demersal crustacean fishery (PUCV-IFOP) in collaboration with the fishing company Camanchaca Pesca Sur, S.A. The Altair I (SFU) and the Trauwun (NFU) trawling vessels were used. Due to the fishing gear (i.e. trawling), there was no minimum commercial size. The captured samples (N = 85) were kept on the vessels in 50 L containers filled with sea water until they were transferred to track and transported the same day of their capture to the Hydrobiological Resources Laboratory of the Universidad Católica de la Santísima Concepción (Concepción, Chile). In the laboratory, the sex and state of maturity of the juvenile squat lobsters was determined using a stereomicroscope (BA-310, Motic). Adaptive differences in the first pair of pleopods were used to determine sex because female pleopods are adapted to incubate eggs, whereas males have modified copulatory organs. The developmental stage of individuals was determined using a standard methodology [66], which describes a white coloration of the gonad with a reduced space within the cephalothorax cavity among immature juveniles. From the total of 85 individuals (44 individuals from the NFU and 41 individuals from the SFU), 30 juvenile females from each collection site (sample site inside each breeding area; n = 60) were selected and frozen at -80°C until further analyses. Juvenile males were not considered in this study because insufficient male samples were obtained for the analyses (n = 9; total = ~90% female vs. ~10% male). In addition, females play a key role in the stability of these areas, specifically through the production of offspring (see: [41,48,66]. The “bioenergetic fuel” or energy reserves (such as lipids and fatty acids) of juvenile females is also particularly interesting for the focus of this study because they store these bioenergetic compounds for the first reproduction event and then transfer them to their first broods.

### Sizes and total dry weight

The cephalothorax length (CL) of each female was measured from the posterior margin of the cephalothorax to the base of the rostral spine. Then, all viscera (i.e., organs from the digestive and reproductive systems) and all muscle (i.e., from the anterior tail) were excised from each juvenile female. We could not separate the different organs due to the condition of the samples (i.e., a very small size), so we decided to designate the set of internal organs as viscera (which are mainly composed of hepatopancreas (~ 90%); stomach and ovary (~10%)) [67,68]. These samples were stored separately in 15 mL centrifuge tubes (i.e., Falcon tubes) and were subsequently lyophilized for 48 h at -70°C to remove any remaining water. Once dry, the samples were weighed to obtain the net weight. In order to obtain the total dry weight of each juvenile female, the weight of viscera and muscle were added to the total dry weight (TDW) of its exoskeleton and body remains. The viscera and muscle samples (20 mg of dry weight) were then used to extract lipids and assess fatty acid profiles, as follows.

### Quantification of lipid content

For the extraction of lipids, 20 mg of the dry weight (DW) of viscera and 20 mg DW of muscle were used. The total lipid analysis was performed using the standard method [69]. This method uses dichloromethane and methanol as organic solvents for lipid extraction. Each sample was suspended in 5 mL of the dichloromethane-methanol solvent (2: 1 v/v) and placed in an ultrasonic bath for 10 min. at room temperature. Then, 4 mL of 0.88% potassium chloride (KCl) solution (in ultra-pure water) was added. The mixture was homogenized and centrifuged at 1500 rpm for 5 min. After centrifugation, two phases were separated, and the lower phase was transferred to a previously weighed 5 mL tube. The solvent was evaporated in a sample concentrator (109A YH-1, Glas-Col) to then weigh the lipid content of the sample by subtracting the weight of the (previously weighed) sample’s tube from the total weight of the tube including the extracted lipids. The data were collected as mg g DW^-1^ and as a percentage of DW (%DW).

### Fatty acid profiles

After analyzing the total lipid content, the fatty acid profiles (i.e., fatty acid methyl esters) were quantified. This was done following the standard method [70] with slight modifications [2], which uses n-hexane as an organic solvent of fatty acids. In brief, in a 9 mL reaction tube, 1 mL of the lipid extract from the previous section was mixed with 2 mL of 1% sulfuric acid and incubated for 3 h at 70°C in a thermoshaker (DBS-001, MRC). After incubation, 3 mL of n-hexane was added to each tube and was vortexed (in a Select Vortexer, model SBS100-2) for 15 s, three times. The supernatant contained in the tube was then transferred to an amber bottle to dry the solvent with nitrogen gas in a sample concentrator (109A YH-1, Glas-Col). Once dry, 1 mL of hexane was added and this sample was transferred to a 1.5 mL amber vial to be injected in a gas chromatograph (7890A, Agilent) mounted with a column to measure fatty acid methyl esters (DB225 column, J&W Scientific, of 30 m length, 0.25 mm intermediate diameter, and a 0.25 μm film). An internal standard (C23: 0, tricosanoic acid) was added to each sample prior to chromatography. All data (size, biomass, lipid content and fatty acids) are shown as average values and standard deviation.

### Statistics

Statistical analyses were based on standard methods [71,72] and were carried out in BRODGAR 2.7.5 (Highland Statistics Ltd.), STATISTICA 8 (StatSoft), PRIMER 6, and + PERMANOVA statistical software [73]. Compliance with the assumptions of homogeneity and normality of variance (P > 0.05) was corroborated, prior to analyses. A general additive model (GAM) was used to explain the differences in sea surface temperatures and chlorophyll-a between the two breading areas (NFU vs SFU) and their variations throughout the year. To perform this analysis, a function based on the R-mgcv package was used [71]. A one-way ANOVA was performed to assess differences in the size and biomass, and two-way ANOVA was performed to assess differences in lipid content, each FA and FA ratios of the two breeding sites (NFU vs. SFU). To multiple comparisons, a post-hoc test (Tukey test) was used. A multivariate analysis was carried out to compare the fatty acid profiles of these two sites. As a first step, a PERMANOVA was used to compare differences in the fatty acid profiles, while a principal coordinate analysis (PCoA) with a Euclidean distance matrix was used to determine the groupings among the samples. Finally, a similarity percentage analysis (SIMPER) was performed to quantify the contribution of each variable to the differences found among groupings.

## Results

### Environmental parameters

**Fig 2:**
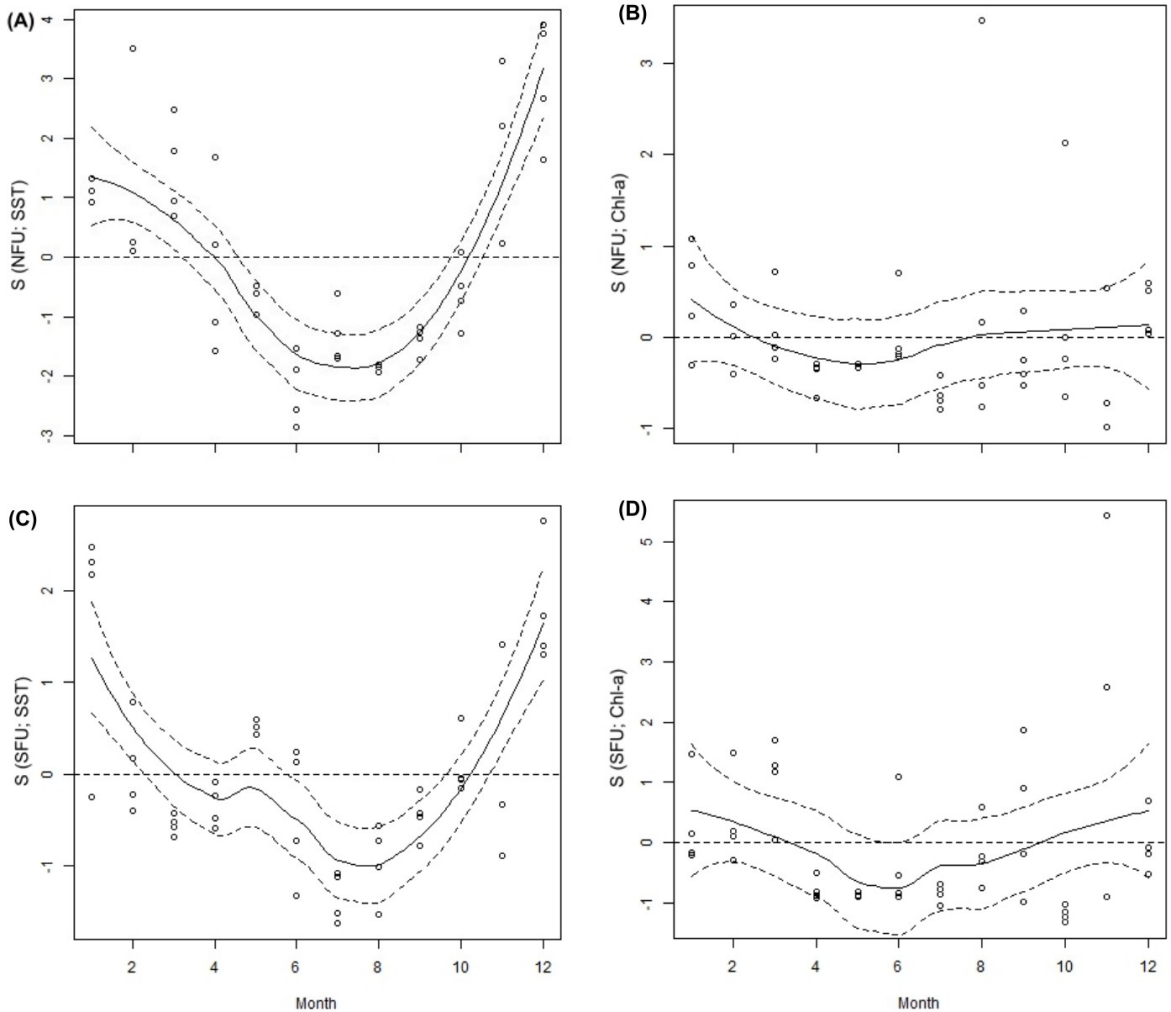
Monthly variation of sea surface temperature (SST) and chlorophyll-a (Chl-a) during 2016 off the coast of Coquimbo and Concepción. Plot (A and B) sea surface temperature (°C), (C and D) chlorophyll-a (mg m^3^). Continuous line: Estimated smoothing function; Segmented line: 95% confidence intervals; dots represent mean values for each month. Months = 12; January to December. NFU, northern fishing unit; SFU, southern fishing unit.

The data from the GIOVANNI website showed a slight, yet noticeable difference between the sea surface temperature (SST) in NFU vs. SFU. In the month of May 2016, the SST in the NFU was 15.62 ± 0.25°C, while it was only 14.84 ± 0.08°C in the SFU. The Chl-a concentration in the NFU was much lower (i.e., 0.7 ± 0.03 mg m^-3^) than that in the SFU (i.e., 0.82 ± 0.04 mg m^-3^) (Table 1). According to the GAM, the smoothing function (S) of SST (Fig 2A and 2B and S1 Table) and Chl-a (Fig 2C and 2D and S2 Table) showed differences between the two breeding areas. In general, during an annual cycle, in contrast to the NFU, the SFU showed lower average SST values and higher average values of Chl-a (Table 1).

**Table 1:** Average sea surface temperature and chlorophyll-a for each month of the year 2016 off the coasts of Coquimbo and Concepción, Chile.

Fishing unit	Environmental variable	Jan	Feb	Mar	Apr	May	Jun	Jul	Aug	Sept	Oct	Nov	Dec
NFU	SST (°C)	18.67 ± 0.2	18.24 ± 1.66	18.38 ± 0.82	16.41 ± 1.46	15.62 ± 0.25	13.79 ± 0.61	14.38 ± 0.51	13.52 ± 0.06	13.72 ± 0.24	14.19 ± 0.57	16.41 ± 1.55	17.2 ± 1.06
	Chl-a (mg m^3^)	1.15 ± 0.61	0.78 ± 0.31	0.97 ± 0.43	0.54 ± 0.17	0.7 ± 0.03	1.16 ± 0.44	0.56 ± 0.16	1.86 ± 1.96	1.13 ± 0.36	1.75 ± 1.25	1.14 ± 0.82	1.19 ± 0.29
SFU	SST (°C)	16.79 ± 1.29	15 ± 0.53	14.17 ± 0.11	14.18 ± 0.23	14.84 ± 0.08	13.72 ± 0.74	12.61 ± 0.28	12.79 ± 0.42	13.09 ± 0.25	13.44 ± 0.35	13.23 ± 1.2	14.76 ± 0.67
	Chl-a (mg m^3^)	1.81 ± 0.79	1.9 ± 0.77	2.62 ± 0.71	0.82 ± 0.19	0.82 ± 0.04	1.38 ± 0.93	0.87 ± 0.15	1.57 ± 0.56	2.19 ± 1.24	0.63 ± 0.12	4.23 ± 3.16	1.87 ± 0.52

NFU, northern fishing unit; SFU, southern fishing unit; SST, sea surface temperature; Chl-a, chlorophyll-a. Mean values ± SD.

### Size and total dry weight of juvenile females

**Fig 3:**
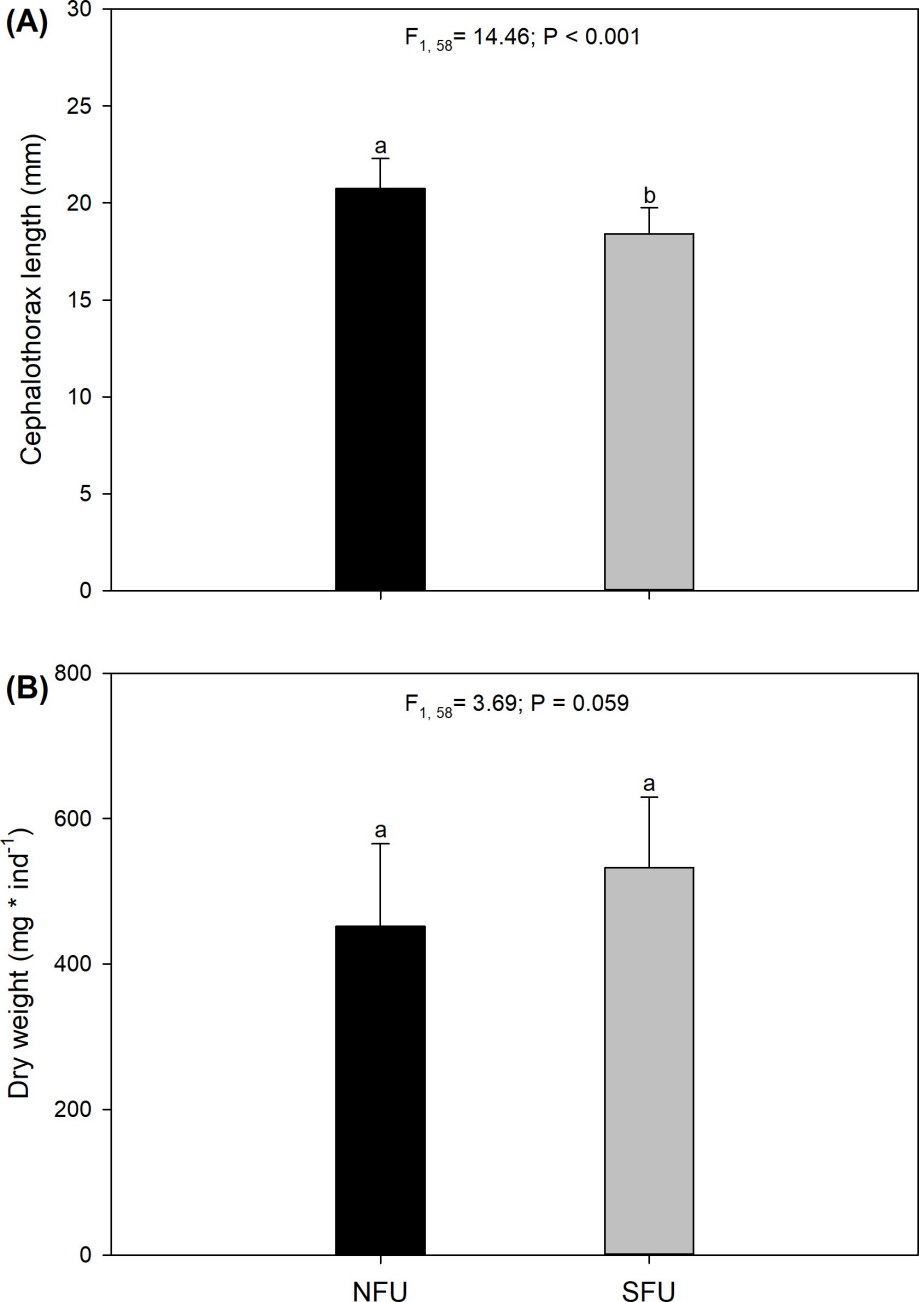
Morphological parameters of juvenile *Pleuroncodes monodon* females from two breeding areas (off the coasts of Coquimbo and Concepción). Plot (A) Cephalothorax length (mm), (B) dry weight (mg ind^-1^). One-way ANOVA was used to compare the morphological parameters. Average values ± S.D. are given. Different letters indicate significant differences. NFU, northern fishing unit; SFU, southern fishing unit.

We found significant differences (F_1, 58_ = 14.46; P < 0.001) between the CL of the females from the NFU and those from the SFU (S3 Table). The CL values of the former were, on average, greater (20.74 ± 1.56 mm) than those of the latter (18.41 ± 1.35 mm; Fig 3A). However, the biomass measured as dry weight did not differ significantly between these two sites (F_1, 58_ = 3.69; P = 0.059; S3 Table), although the juvenile females from the NFU had slightly lower weights (451.8 ± 113.4 mg) than those from the SFU (532.4 ± 97.2 mg; Fig 3B).

### Lipid content

**Fig 4:**
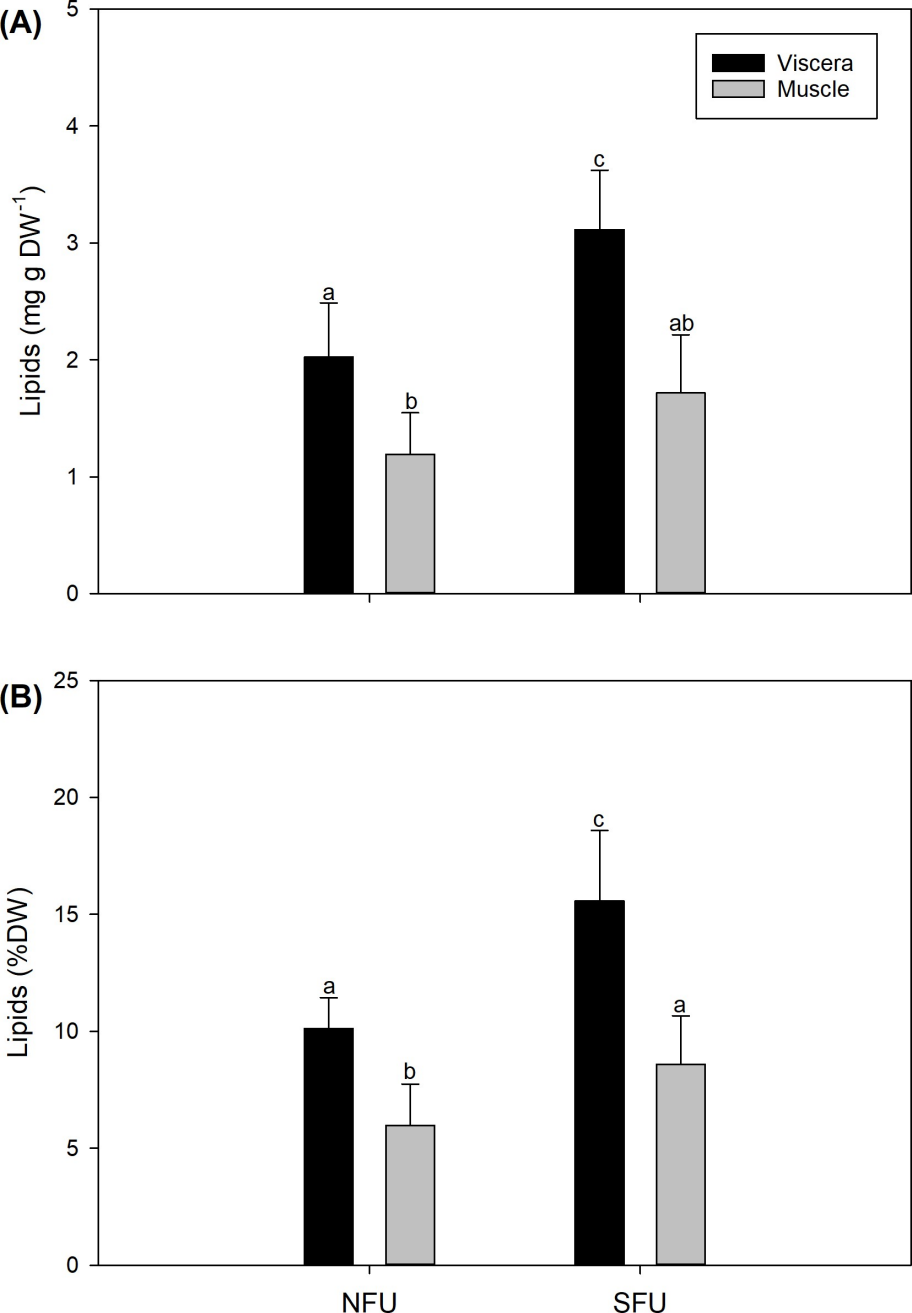
Lipid content of the visceral and muscle of juvenile *Pleuroncodes monodon* females from two breeding areas (off the coasts of Coquimbo and Concepción). Plot (A) lipid content (mg g DW^-1^), (B) lipid percentage (% DW). Two-way ANOVA was used to compare the lipid content. Average values ± S.D. are given as (A) the absolute values (mg g DW^-1^) and (B) the percentage of the mass (% DW). Different letters indicate significant differences. NFU, northern fishing unit; SFU, southern fishing unit.

The total lipid content of juvenile females (F_1, 116_ = 52.18; P < 0.0001) were significantly different in the two locations. The viscera and muscle also showed significant differences (F_1, 116_ = 109.87; P < 0.0001), but no significant difference was found in their interaction (F_1, 116_ = 0.25; P = 0.778) (S3 Table). The viscera of the females from the SFU had a higher lipid content (3.11 ± 1.08 mg) compared to those from the NFU (2.02 ± 0.66 mg), while for the muscle, the lowest lipid content was found in the females from the NFU (1.19 ± 0.55 mg) than in the females from the SFU (1.71 ± 0.81 mg) (Fig 4A).

Regarding the percentage of lipids in relation to DW, significant differences were found when comparing locations (F_1, 116_ = 36.74; P < 0.0001) and viscera and muscle (F_1, 116_ = 74.5; P < 0.0001), but no significant differences were observed in their interaction (F_1, 116_ = 0.037; P = 0.847) (S3 Table). The percentage values of lipids were similar to those of absolute values of lipid content. The viscera of the juvenile females of the SFU had a higher percentage of lipids (15.56 ± 5.42% DW) than those from the NFU (10.11 ± 3.32% DW), while for the muscle, the lowest percentage of lipids were detected in the juvenile females from the NFU (5.96 ± 2.76% DW) than in the females from the SFU (8.58 ± 4.07% DW) (Fig 4B).

### Fatty acid profiles

A total of 31 different fatty acids (FAs) were found in the muscle and viscera of the juvenile females from the NFU (i.e., 30 FA) and from the SFU (i.e., 29 FA) (Table 2). Among the FAs of juvenile females from the NFU, the viscera had 30 FAs and thus the highest amounts of total FAs in our study. These FAs included: 13 saturated fatty acids (SFAs), eight monounsaturated fatty acids (MUFAs), five polyunsaturated fatty acids ω6 (ω6-PUFAs), and four ω3-PUFAs, with particularly high concentrations of palmitic acid (C16: 0). While in the muscle of these females, a total of 20 FAs were found, including: 10 SFAs, three MUFAs, four ω3-PUFAs, and three ω6-PUFAs, with high concentrations of C16: 0 acid, oleic acid (C18: 1ω9), and docosahexaenoic acid (DHA, C22: 6ω3). In contrast, 29 FAs were found in the viscera of these juvenile females from the SFU, comprising: 12 SFAs, eight MUFAs, five ω6-PUFAs, and four ω3-PUFAs, with particularly high concentrations of C16: 0, C18: 1ω9, and DHA. In addition, 23 different FAs were found in the muscle of juvenile females from the SFU: 12 SFAs, six MUFAs, two ω6-PUFAs, and three ω3-PUFAs, with particularly high concentrations of C16: 0, and c18: 1ω9 acids (Table 2). Among all analyzed samples, the FA with the highest concentration was C16: 0, reaching its highest levels in the viscera of juvenile females from the SFU (i.e. off the coast of Concepción).

**Table 2:** Fatty acid profiles of the viscera and muscle of juvenile *Pleuroncodes monodon* females from two breeding areas (off the coasts of Coquimbo and Concepción).

	mg g DW^-1^			
Fatty acids	NFU		SFU	
	Viscera	Muscle	Viscera	Muscle
C8:0	0.53 ± 0.06^a^ (1.1)	0.41 ± 0.05^b^ (2.08)	0.54 ± 0.04^a^ (0.95)	0.48 ± 0.03^ab^ (1.44)
C10:0	ND	ND	1.01 ± 0.44 (1.77)	0.44* (1.32)
C11:0	0.51 ± 0.07^a^ (1.05)	0.43 ± 0.06^a^ (2.22)	1.11 ± 0.48^b^ (1.93)	1.11 ± 0.66^b^ (3.3)
C12:0	0.52 ± 0.05^a^ (1.08)	0.83* (4.23)	0.75 ± 0.17^b^ (1.32)	0.89 ± 0.31^b^ (2.66)
C13:0	0.62 ± 0.06^a^ (1.27)	0.68 ± 0.09^a^ (3.48)	0.53 ± 0.08^a^ (0.92)	0.88 ± 0.28^a^ (2.61)
C14:0	1.47 ± 0.28^a^ (3.04)	0.58 ± 0.09^b^ (2.98)	1.96 ± 0.24^a^ (3.43)	0.57 ± 0.09^b^ (1.71)
C15:0	0.66 ± 0.09^a^ (1.36)	0.37 ± 0.01^b^ (1.92)	0.79 ± 0.07^a^ (1.38)	0.5 ± 0.06^a^ (1.49)
C16:0	7.08 ± 1.13^a^ (14.67)	3.28 ± 0.27^b^ (16.8)	9.11 ± 1.29^a^ (15.93)	3.86 ± 0.33^b^ (11.5)
C17:0	0.69 ± 0.07^a^ (1.43)	0.4 ± 0.02^b^ (2.06)	1.12 ± 0.14^c^ (1.96)	0.51 ± 0.16^ab^ (1.53)
C18:0	2.21 ± 0.2^a^ (4.58)	1.33 ± 0.11^b^ (6.81)	2.64 ± 0.29^c^ (4.62)	1.29 ± 0.16^b^ (3.85)
C20:0	0.45 ± 0.09 (0.93)	ND	ND	ND
C22:0	0.35 ± 0.001 (0.73)	ND	ND	ND
C23:0	2.09 ± 0.46^a^ (4.33)	ND	3.63 ± 1.29^a^ (6.35)	7.66* (22.8)
C24:0	0.23* (0.47)	0.35* (1.81)	2.9* (5.07)	1.33* (3.97)
**Σ FAS**	**17.41 ± 0.25**^**a**^ **(36.04)**	**8.66 ± 0.12**^**b**^ **(44.39)**	**26.09 ± 0.35**^**c**^ **(45.63)**	**19.52 ± 0.14**^**d**^ **(58.18)**
C14:1ω5	0.66 ± 0.1^a^ (1.37)	0.47 ± 0.05^b^ (2.4)	0.55 ± 0.13^ab^ (0.96)	0.58 ± 0.06^a^ (1.73)
C15:1	0.72 ± 0.11^a^ (1.48)	ND	0.8 ± 0.11^a^ (1.39)	0.86 ± 0.4^a^ (2.57)
C16:1ω7	1.25 ± 0.38^a^ (2.58)	0.52 ± 0.05^b^ (2.66)	3.07 ± 0.51^c^ (5.37)	0.98 ± 0.14^a^ (2.91)
C17:1	1.29 ± 0.16^a^ (2.68)	ND	1.14 ± 0.11^a^ (1.99)	0.59 ± 0.02^b^ (1.77)
C18:1ω9	3.29 ± 0.5^a^ (6.82)	1.58 ± 0.24^b^ (8.12)	6.78 ± 0.75^c^ (11.85)	3.12 ± 0.2^a^ (9.28)
C20:1ω9	1.97 ± 0.73^a^ (4.09)	ND	1.33 ± 0.11^a^ (2.33)	0.77* (2.29)
C22:1ω9	1.01 ± 0.29^a^ (2.09)	ND	0.88 ± 0.07^a^ (1.55)	ND
C24:1ω9	1.09 ± 0.3^a^ (2.26)	ND	1.1 ± 0.27^a^ (1.92)	ND
**Σ MUFA**	**11.28 ± 0.18**^**a**^ **(23.37)**	**2.57 ± 0.16**^**b**^ **(13.18)**	**15.65 ± 0.26**^**c**^ **(27.36)**	**6.9 ± 0.18**^**d**^ **(20.55)**
C18:2ω6t	0.83 ± 0.34^a^ (1.72)	0.48 ± 0.1^b^ (2.47)	0.63 ± 0.08^ab^ (1.1)	ND
C18:2ω6c	0.9 ± 0.18^a^ (1.86)	0.43 ± 0.01^b^ (2.22)	0.95 ± 0.05^a^ (1.67)	0.67 ± 0.28^ab^ (2)
C18:3ω6	1.53 ± 0.29^a^ (3.16)	0.76 ± 0.2^b^ (3.88)	1.46 ± 0.16^a^ (2.55)	ND
C20:2ω6	1.02 ± 0.24 (2.12)	ND	0.39* (0.68)	ND
C20:3ω6	2.4 ± 1.47^a^ (4.97)	1.33 ± 0.11^a^ (6.81)	0.83 ± 0.08^b^ (1.44)	0.37 ± 0.11^c^ (1.1)
**Σ PUFA ω6**	**6.68 ± 0.18**^**a**^ **(13.83)**	**3 ± 0.12**^**b**^ **(15.38)**	**4.26 ± 0.07**^**c**^ **(7.44)**	**1.04 ± 0.15**^**d**^ **(3.1)**
C18:3ω3	1.34 ± 0.55^a^ (2.77)	ND	0.64 ± 0.08^b^ (1.12)	ND
C20:3ω3	3.11 ± 0.52^a^ (6.44)	2.02 ± 0.32^b^ (10.35)	2.65 ± 0.35^ab^ (4.63)	1.36 ± 0.27^c^ (4.04)
C20:5ω3 (EPA)	2.71 ± 0.6^a^ (5.61)	1.62 ± 0.21^b^ (8.33)	3.55 ± 0.39^ac^ (6.21)	2.41 ± 0.27^ad^ (7.18)
C22:6ω3 (DHA)	5.77 ± 1.9^a^ (11.95)	1.63 ± 0.25^b^ (8.36)	4.34 ± 0.52^a^ (7.6)	2.34 ± 0.3^c^ (6.97)
**Σ PUFA ω3**	**12.93 ± 0.78**^**a**^ **(26.77)**	**5.27 ± 0.15**^**b**^ **(27.04)**	**11.18 ± 0.28**^**c**^ **(19.56)**	**6.11 ± 0.18**^**d**^ **(18.19)**
**Σ PUFA**	**19.61 ± 0.58**^**a**^ **(40.6)**	**8.27 ± 0.13**^**b**^ **(42.42)**	**15.44 ± 0.23**^**c**^ **(27)**	**7.15 ± 0.17**^**d**^ **(21.29)**
**Σ FA**	**48.3 ± 0.19**^**a**^ **(100)**	**19.50 ± 0.08**^**b**^ **(100)**	**57.8 ± 0.16**^**c**^ **(100)**	**33.57 ± 0.09**^**d**^ **(100)**
C16:0/C18:0	3.26 ± 0.4^a^	3.06 ± 0.55^a^	4.7 ± 0.72^b^	5.13 ± 0.67^b^
DHA/EPA	2.9 ± 1.13^a^	1.03 ± 0.04^b^	1.42 ± 0.12^a^	0.99 ± 0.06^b^
PUFA/SFA	0.85 ± 0.15^a^	0.91 ± 0.2^a^	0.82 ± 0.09^a^	0.79 ± 0.07^a^

NFU, northern fishing unit; SFU, southern fishing unit; ND, not detected; SFA, saturated fatty acids; MUFA, monounsaturated FA; PUFA, polyunsaturated FA; EPA, eicosapentaenoic acid; DHA, docosahexaenoic acid. Σ SFA = sum of C8:0, C10:0, C11:0, C12:0, C13:0, C14:0, C15:0, C16:0, C17:0, C18:0 C20:0, C22:0, C23:0 and C24:0; Σ MUFA = sum of C14:1ω5, C15:1, C16:1ω7, C17:1, C18:1ω9, C20:1ω9, C22:1ω9 and C24:1ω9; Σ PUFA ω6 = sum of C18:2ω6t, C18:2ω6c, C18:3ω6, C20:2ω6 and C20:3ω6; Σ PUFA ω3 = sum of C18:3ω3, C20:3ω3, C20:5ω3 and C22:6ω3; PUFA = sum of ω3 and ω6 PUFAs; Σ FA = sum of Σ SFA, Σ MUFA, and Σ PUFA. Asterisks indicate only one FA sample (unavailable SD). Mean values ± SD, n = 120. Different superscript letters indicate significant differences. The percentage is indicated in parentheses.

Two-way ANOVA was performed.

**Fig 5:**
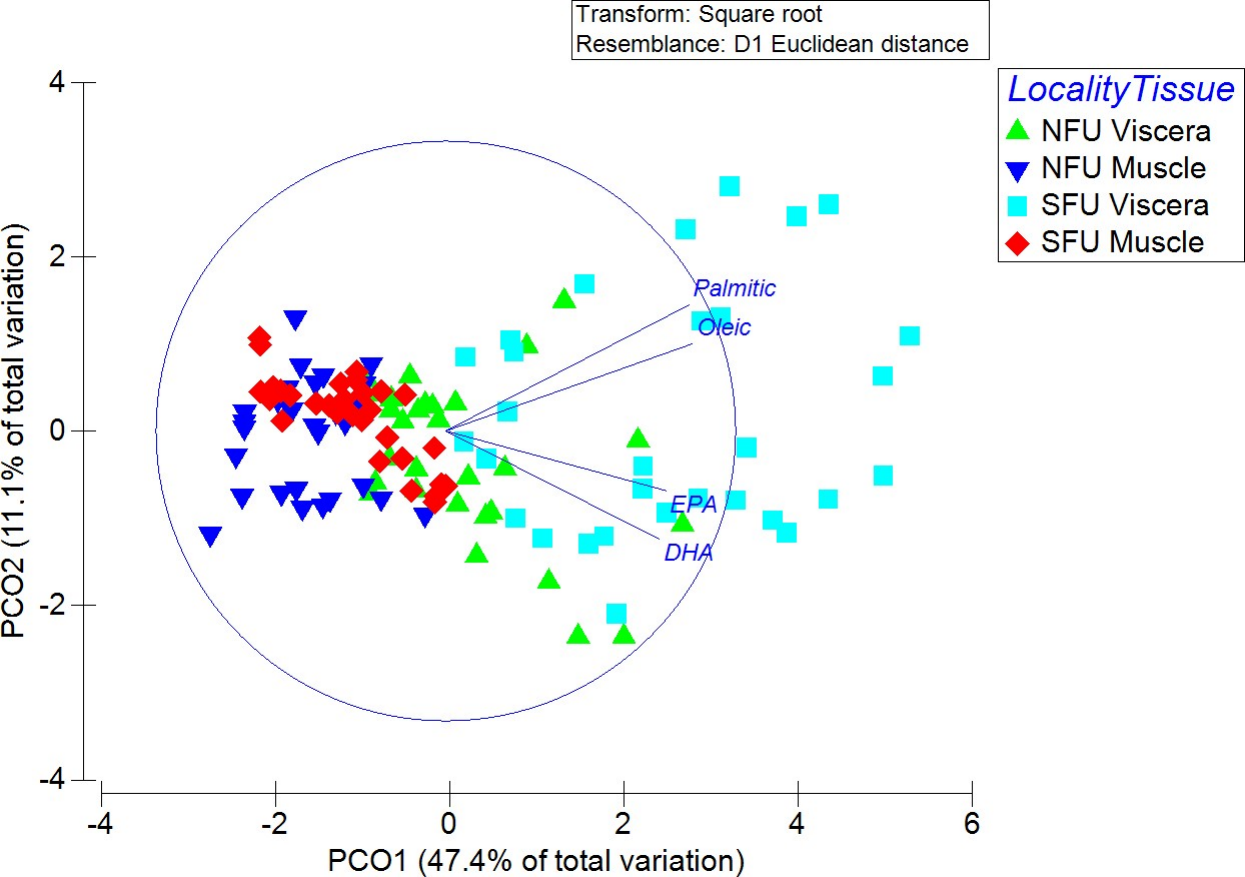
Principal coordinate analysis (PCoA) of the fatty acid profiles of the visceral and muscle of juvenile *Pleuroncodes monodon* females from two breeding areas (off the coasts of Coquimbo and Concepción). The figure illustrates the fatty acids with the highest contributions. NFU, northern fishing unit; SFU, southern fishing unit.

When comparing the FA profiles of the two localities and the viscera versus the muscle, clear significant differences were observed in the viscera versus the muscle (PERMANOVA; Pseudo-F = 26.13; P <0.001), in the two “localities” (PERMANOVA; Pseudo-F = 53.51; P < 0.001), and in their interaction (PERMANOVA; Pseudo-F = 13.67; P <0.001). When analyzing the interaction, the PCO1 axis explained 47.4% of the variance, while the PCO2 axis explained 11.1% of the variance. Although there was no clear separation of the different groups, the viscera of females from the SFU had a higher dispersion, which separated them from the rest of the groups (Fig 5). When analyzing the locality factor, there was a slight separation between the two localities, but when evaluating the organs factor, different fatty acids were clearly observed in the two organs (Fig 5).

The SIMPER analysis revealed that the FAs with the greatest contribution to groupings by location (i.e., NFU vs. SFU) were palmitic acid (45.34%), oleic acid (19.61%), and DHA (10.93%) (S4 Table). The same FAs were the main contributors to groupings by viscera and muscle, as follows: palmitic acid (42.7%), oleic acid (23.7%), and DHA (9.43%) (S5 Table).

### Fatty acid ratios

The ratio of C16:0/C18:0 SFAs only showed significant differences between locations (F_1, 92_ = 21.052; P < 0.0001), while there were no significant differences between viscera and muscle (F_1, 92_ = 0.098; P = 0.755), nor in their interaction F_1, 92_ = 0.142; P = 0.707) (Table 2). The highest proportion of these SFAs was found in the muscle of juvenile females from the SFU (5.13 ± 0.67), followed by that in their viscera (4.7 ± 0.72), while the lowest proportions were found in the muscle (3.06 ± 0.55) and viscera (3.26 ± 0.4) of juvenile females from the NFU (Table 2).

The ratio of DHA/EPA PUFAs only showed significant differences between the viscera and muscle (F_1, 80_ = 20.66; P < 0.0001), but not when comparing locations (F_1, 80_ = 1.407; P = 0.239), nor the interaction of these factors (F_1, 80_ = 0.899; P = 0.346) (Table 2). The viscera of the juvenile females from the NFU had the highest ratios (2.9 ± 1.13), followed by the viscera of the juvenile females from the SFU (1.42 ± 0.12). On the contrary, the muscle of juvenile females from the NFU (1.03 ± 0.04) and the SFU (0.99 ± 0.06) presented the lowest values of this ratio (Table 2).

The ratio of PUFAs/SFAs was not significantly different in the two locations (F_1, 84_ = 0.35; P = 0.55), nor when comparing the viscera and muscle (F_1, 84_ = 0.02; P = 0.9), nor in the interaction of these factors (F_1, 84_ = 0.03; P = 0.87) (Table 2). Despite, these ratios being overall quite similar, the highest values were found in the muscle (0.91 ± 0.2) and viscera (0.85 ± 0.15) of the juvenile females from the NFU, followed by the viscera (0.82 ± 0.09) and muscle (0.79 ± 0.07) of the juvenile females from the SFU (Table 2).

## Discussion

The red squat lobster is one of the principal demersal crustacean fishery resources in the Humboldt Current System (HCS). We analyzed the lipid content and fatty acid profiles as a proxy of the bioenergetic state of juvenile female red squat lobsters from two extraction units that are far apart, yet both off the Chilean coast: a) the northern fishing unit (NFU) and b) the southern fishing unit (SFU), which both have nearby breeding areas [40]. Differences in size, lipid content, and fatty acid profiles were found between these sites. Additionally, within the respective breeding areas, we found differences in the proportion of saturated fatty acids (C16:0/C18:0) and essential fatty acids (DHA/EPA). Our findings may be the result of a phenotypic plastic response or local adaptation to the environmental conditions which *P*. *monodon* experiences throughout its developmental stages [26,27,48,53,56,59].

During its annual cycle, the Humboldt Current System is characterized not only by marked seasonal changes in its environmental parameters (SST and Chl-a concentrations), but also by differing physico-chemical and climatic conditions at small spatial scales [17–19]. The northern region is characterized by continuous upwellings, while in the southern region upwellings are more seasonal [17,19]. Consequently, marine organisms from northern Chile have greater food availability, while those marine organisms living south of 30°S experience seasonal peaks in food availability, which may lead to the development of more efficient techniques of energy intake and storage among individuals in the SFU compared to those in the NFU [74]. Therefore, differences in the environmental conditions experienced by juvenile females prior to their capture in the two breeding areas may reflect changes in their bioenergetic reserves (measured as the lipid content and fatty acid composition in the present study).

Additionally, we found differences in the length of the cephalothorax, which was, on average, longer among juvenile females from the NFU than those from the SFU. There is ample literature that supports an increase in body size with latitude [24,75–77]. However, in our study, we did not find such a positive and directly proportional relationship between body size and latitude. SST could regulate growth rates, causing the juvenile females in the north to grow faster than those in the south [78]. Nevertheless, the body weight (measured as total biomass) was similar among juvenile females from the NFU and the SFU. The SFU experiences marked and intense upwelling periods with higher primary production and a large contribution of organic matter from freshwater discharge [17]. This higher productivity could generate a diet richer in nutrients (or simply greater amounts of food) and consequently an increase in the biomass of the juvenile females in the SFU. Hence, while the juvenile females in the NFU have a larger size (i.e., cephalothorax length), they have less biomass (i.e., total dry weight) than juvenile females from the SFU. A latent “generational” effect of intense fishing pressure on a resource has been observed to cause a decrease in the average size of individuals (for concept see [79]. In the case of *P*. *monodon*, a higher fishing pressure has been “historically” reported in the SFU as compared to the NFU [41]. This latent effect could explain the smaller average size registered in juvenile SFU females (present study). A similar trend has also been described in widely distributed decapod species that are under intense fishing pressure (*Aristaeomorpha foliacea*, *Aristeus antennatus*, *Crangon crangon*) [80,81].

At low temperatures, organisms tend to accumulate greater energy reserves due to decreasing metabolic rates [23,57,82]. Furthermore, lipids are the main energy source of many marine invertebrates [33,83] and studies have documented that several crustacean species (*P*. *monodon*, *Crangon crangon*, *Meganyctiphanes norvegica*) tend to accumulate greater energy reserves at lower temperatures or during cold seasons [26,27,39,48,84,85]. Along these lines, the juvenile females from the SFU, where lower SSTs are the norm, had higher lipid content than those from the breeding ground in the NFU, where there are higher SST. Hence, females in the SFU may accumulate greater energy reserves as a result of lower SST in order to maintain metabolic functions despite low temperatures [82].

The composition and concentration of fatty acids is vital for the nutrition and functioning of animals [86]. The highest content of fatty acids (mainly essential) is usually found in the hepatopancreas and/or viscera, which is the main organ that stores these bioenergetic compounds, whereas the muscle stores mainly fatty acids of the structural type [31,33,57,87,88]. This may explain why we measured overall higher concentrations of total fatty acids in the viscera, as opposed to the muscle of juvenile females. Additionally, the highest concentration of fatty acids was found in the SFU, most likely due to the fact that juvenile females in this region may experience greater food availability linked to more intense (though seasonal) upwellings [17,19]. Also, the water temperature may influence the total content of fatty acids due to its importance in the fluidity of the membranes [39]. This may force the juvenile females from the SFU to develop more efficient pathways to accumulate bioenergetic reserves to be used during periods of food shortage. Bascur and collaborators [27] found a similar tendency, where at cold winter temperatures, the red squat lobster accumulated greater bioenergetic reserves to tolerate periods of low food availability.

Crustaceans are known to generally accumulate large amounts of polyunsaturated fatty acids [86]. In our study, opposed to expectations, the polyunsaturated fatty acids were more abundant among females from the NFU. This accumulation should be greater in austral regions, since polyunsaturated fatty acids can improve the functions of membranes at low temperatures [39], but our results indicated an opposite trend. We argue that the reason for a lower amount of polyunsaturated fatty acids and a higher amount of saturated fatty acids in juvenile females from the SFU compared to those from the NFU, is most likely related to: i) weak metabolic capacity of juveniles to biosynthesize PUFAs from precursor fatty acids (e.g. SFA = C16: 0 Palmitic; MUFA = C:18 1n9 Oleic) (for concept on metabolism and functions of lipids and fatty acids see: [89,90], ii) the type of food or prey. The prey in the SFU may strongly differ from that in the NFU due to the greater discharge of fresh water in the southern region coming from the large number of river inlets and higher rainfall. The continental shelf is also wider in the SFU zone and this area is shallower, causing a greater recirculation of nutrients (e.g. organic matter) in the water column [19,74]. This could allow for a greater productivity and higher nutrient availability for benthic organisms, thus producing a higher amount of organic matter and saturated fatty acids of terrestrial origin to be found in the sea around the SFU [91,92]. This could hence induce a higher SFA content in southern females compared to northern females. Although the influence of organic matter of terrestrial origin can be estimated by analyzing the profile of fatty acids as trophic biomarkers [91,93], for a more detailed analysis of biomarker levels of terrestrial material, we recommend complementing these analyses with sterol quantifications because their decomposition or transformation is slower and more precise over time [92,94].

Additionally, it is highly likely that juvenile females of the red squat lobster cannot, or are barely able to, biosynthesize large amounts of polyunsaturated fatty acids, as is the case among other marine decapod crustaceans [31]. Juvenile females from the SFU breeding ground proved to have a diet rich in saturated fatty acids (most likely from terrestrial origin), while those from the NFU breeding ground received a diet almost exclusively of marine origin, with large amounts of polyunsaturated fatty acids [95–97]. Among the classes of fatty acids, palmitic, oleic, DHA and EPA fatty acids contributed most to the similarity between breeding areas and the analyzed organs (S4 and S5 Tables). High amounts of palmitic acid, oleic acid, DHA and EPA have also been documented in the hepatopancreas and muscle of juvenile *Portunus trituberculatus* [31] and *Scylla serrata* [98].

The assessment of the ratios of certain fatty acids in marine organisms is a useful technique to reconstruct trophodynamic links within marine ecosystems; hence, these ratios serve as trophic biomarkers [99]. For example, the polyunsaturated fatty acids DHA and EPA are not only extremely important for the physiological processes involved in the growth and reproduction of marine species, they are also well-established trophic biomarkers [100]. EPA, for instance, controls the proper functioning of vascular systems and is an essential component in the structure of cell membranes [101], while DHA is a biomolecule of great importance in processes in the brain and the nervous system, as well as an essential component of the retina of several species [102]. In our study, the saturated fatty acids C16: 0 (palmitic acid) and C18: 0 (stearic acid) were the main fatty acids found in both the viscera and muscle of juvenile females from both the NFU and the SFU. These fatty acids have also been commonly found in the tissues of other crustacean species [103–105]. Regarding the ratios, we found significant differences between locations for C16:0/C18:0 ratio, for DHA/EPA ratios only found significant differences between organs and no significant differences were detected for the PUFA/SFA ratio. These differences, which were found in both locations, could reflect strategies to physiologically balance the proportions of fatty acids in order to maintain proper functioning (i.e., homeostasis) of the organisms at different temperatures [82]. In general, the three ratios we calculated (i.e. C16:0/C18:0, DHA/EPA and PUFA/SFA ratios) were highly similar between localities and between the viscera and muscle. Hence, we propose that by adjusting the ratios between the types/classes of fatty acids, the organisms may be able to compensate for the differences we found in the fatty acid profiles between both sites, especially regarding the amount of polyunsaturated fatty acids in relation to the temperature of their respective breeding grounds. Consequently, the levels and variations in lipid content and fatty acid profiles may be the result of physiological adaptations to different environmental conditions [33]. Also, we could infer that according to the fatty acid profiles found in the present study and in concordance with Lovrich and Thiel [40], the juvenile females of *P*. *monodo*n are filter feeders that most likely feed on phytoplankton and zooplankton. In our study, the fatty acid profiles, which serve as "dietary markers" or "trophic biomarkers," indicated that juveniles may feed on phytoplankton (16: 1ω7 + C16 PUFA + 20: 5ω3; C18 PUFA + 22: 6ω3), zooplankton (copepods: 20: 1ω9 + 22: 1ω11), and detritus (18: 1ω9). Considering that in most crustaceans these PUFAs cannot be biosynthesized (due to the lack of desaturase activities; [106]), they are obtained directly from food and have been widely used as fatty acid trophic markers in marine environments [38,91,92,107,108].

In fish nutrition studies, the DHA/EPA ratio has been well-documented [109]. However, in juvenile crustaceans, there are few publications that address this ratio (see Table 3). The DHA/EPA ratio found in the muscle of *P*. *monodon* is relatively similar to that found in marine decapods such as *Paralithodes platypus* and *S*. *olivacea*, while the ratio found in the viscera of *P*. *monodon* is similar to *P*. *trituberculatus* and *S*. *serrata* from coastal habitats (see Table 3). The DHA/EPA ratio proved to be higher in the viscera (e.g. hepatopancreas) than in the muscle, which reflects a greater accumulation of DHA than EPA. For a global comparison of this ratio, in Table 3 we included the values of the DHA/EPA ratio of various species of juvenile decapod crustaceans distributed throughout different geographical regions of the planet. Although, in an overview there is no marked trend, the registered values of the DHA/EPA ratio are probably not only linked to fixed and characteristic traits of each family of crustacean (phylogenetic), but also to their range of geographic distribution, type of aquatic habitat (marine, estuarine, freshwater), and the climatic characteristics of these regions. In general, marine invertebrates that live in temperate and cold regions with predictable productivity present a higher synthesis and ratio of DHA/EPA than those that inhabit hot and tropical regions [95,102]. This could permit a greater flexibility of the cell membranes and tissues in cold environments, as well as aid in the successful development and increased survival in periods of low temperatures and food shortages during winter periods [31,39]. In turn, for freshwater invertebrates, a higher DHA/EPA ratio may be necessary to maintain the functionality of the plasma membrane at extreme hot and cold temperatures [110], and also to support the high energy cost produced by successive spawning events and direct development (e.g. see the case of the shrimp, *Neocaridina davidi* [111]. Future studies could evaluate whether there is a relationship in decapods between phylogeny, habitat type (e.g. marine, estuarine, freshwater) and the value of the DHA/EPA ratio, and whether this ratio could be used as a condition index of decapod crustaceans from different regions and aquatic habitats.

**Table 3:** DHA/EPA ratio of juvenile decapod crustacean species living in different regions (i.e. distribution range).

Species	DHA/EPA ratio	Organ analyzed	Distribution range	Reference
*Pleuroncodes monodon*	1.42–2.9 0.99–1.03	Viscera Muscle	Humboldt Current System (7° S– 37° S)	Present study
*Paralithodes platypus*	~ 1.1	Whole body	Bering Sea (66° N– 36° N)	[112]
*Homarus americanus*	0.63–0.82*	Whole body	Atlantic coast (60° N– 35° N)	[113]
*Maja brachydactyla*	0–52–0.56*	Body without carapace	East coast of the Atlantic ocean (55° N– 44° N)	[114]
*Palaemonetes zariquieyi*	~ 0.38	Whole body	Freshwater, Iberian Peninsula coast (41° N– 38° N)	[115]
*Neocaridina davidi*	~ 3.40 ~ 2.66	Cephalothorax Pleon	Freshwater; native of Asia (24° N– 22° N)	[116]
*Portunus trituberculatus*	1.36–1.60 0.88–0.96	Hepatopancreas Muscle	Asian coastal waters (41° N– 37° S)	[88]
*Portunus trituberculatus*	1.78–1.83 0.78–0.88	Hepatopancreas Muscle	Asian coastal waters (41° N– 37° S)	[117]
*Portunus trituberculatus*	0.91–1.27 0.66–0.90	Hepatopancreas Muscle	Asian coastal waters (41° N– 37° S)	[31]
*Portunus pelagicus*	~ 0.64	Whole body	Estuarine, Indo west Pacific (41° N– 37° S)	[118]
*Lysmata seticaudata*	0.61–3.42	Whole body	Western Atlantic coast (37° N– 32° S)	[119]
*Scylla serrata*	~ 0.86* ~ 1.48* ~ 0.72* ~ 0.64*	Muscle Hepatopancreas Anterior gills Posterior gills	Estuarine, Indo-Pacific coast (34° N– 34° S)	[98]
*Scylla serrata*	1.19–1.95*	Gill	Estuarine, Indo-Pacific coast (34° N– 34° S)	[87]
*Scylla olivacea*	0.75–1.56	Whole body	Indo west pacific (34° N– 34° S)	[120]
*Panulirus cygnus*	~ 0.8	Whole body	Australian coast (22° S– 34° S)	[109]

Asterisks indicate that the values were calculated from the recorded data of the concentration of DHA and EPA.

Although bioenergetic differences were found in benthic juveniles from the two fishing units (present study), a more in-depth and detailed analysis is required to know if these two units (NFU, SFU) correspond to different local populations, metapopulations or a single large population (for concept see: [12,121]). Considering that *P*. *monodon* presents a planktonic larval phase in its biphasic life cycle with five zoea stages: [43,55], and eventually a “high” larval dispersal potential [15], the degree of larval dispersion and natural connectivity between fishing units is still unknown [41]. Moreover, due to the biogeographic break that exists in the middle of both studied populations (30°S) (see: [122,123]), the probability that these populations are genetically connected, as reported in other species of marine invertebrates with biphasic life cycle [123–125], remains uncertain. To confirm the population connectivity of these two groups of squat lobsters, it would be necessary to carry out population genetic structure studies to determine possible relationships between these populations using molecular markers (for concept see [124,126,127]).

Lipids and fatty acids are the bioenergetic fuel of juvenile specimens and can thus affect the quality and survival of future recruits in the exploitable stock of a fishery resource. Bascur and collaborators found that these bioenergetic constituents do not only vary in the tissue of adult female red squat lobsters [26], but also in their juvenile stages (present study). As a carry-over effect, the nutritional status of juvenile females may affect the nutritional status of later stages (i.e., adult phase) and could consequently modify the organoleptic characteristics of adult individuals, which are exploited and commercialized by fishing activities. Currently, only the tail muscle of the red squat lobster is consumed, but the high content of fatty acids we found in their viscera may add value to this fishery resource and advocate for the usage of other organs with high nutritional value [104,128]. Moreover, increased knowledge regarding the spatial variations of these bioenergetic reserves (i.e., lipids and fatty acids), in addition to other biochemical parameters (e.g. glycogen, proteins, amino acids, digestive enzyme) of juvenile populations, could help predict the “bioenergetic condition” (for concept, see [129]) and nutritional quality of future exploitable populations. Within an ecosystem approach, this data could also aid in assigning catches to certain populations (i.e., fisheries traceability) and developing strategies of exploitation and sustainable management, especially for areas where this resource has larger sizes and better nutritional quality.

## Conclusion

The red squat lobster is an important resource for fisheries in the Humboldt Current System, where it supports one of the largest crustacean fisheries in Chile. Juvenile squat lobsters seem to adjust their bioenergetic reserves in relation to the environmental parameters (such as temperature and chlorophyll-a) of their location. On average, these southern juvenile females (from the SFU off the coast of Concepción) had higher lipid and fatty acid content than those inhabiting the NFU. In addition, the viscera had a higher lipid content than the muscle, regardless of the location. Lastly, unlike our expectations, the juvenile females from the NFU had a higher content of polyunsaturated fatty acids than those from the SFU. This could be attributed to differences in the food sources of the two regions, and thus the metabolic capacity to biosynthesize and store essential fatty acids. While the C16: 0/C18:0 ratio was higher in the SFU than in the NFU, the DHA/EPA ratio showed no differences between the two breeding areas. Our resulting data is important for the management of this fishery resource and for the maintenance of healthy adult populations in both fishing grounds, since the studied specimens represent the recruits of future adult populations exploited by the fishing industry. Furthermore, we advocate for the incorporation of bioenergetic parameters in fishery models for recruitment and stock assessment within an ecosystem approach.

## Supporting information

S1 Table Generalized additive modelling of sea surface temperatures (SSTs) during an annual period (January to December of 2016) off the coast of Coquimbo and Concepción, Chile. (DOCX)

S2 Table Generalized additive modelling of chlorophyll-a (Chl-a) during an annual period (January to December of 2016) off the coast of Coquimbo and Concepción, Chile. (DOCX)

S3 Table Statistics (one-way ANOVA for cephalothorax length, dry weight and lipid content, and two-way ANOVA for fatty acids and fatty acid ratios) of the differences in female parameters of viscera and muscle of juvenile *Pleuroncodes monodon* from two breeding areas (off the coasts of Coquimbo and Concepción). (DOCX)

S4 Table Analysis of the percentage of similarity (SIMPER) in fatty acids of juvenile *Pleuroncodes monodon* females from two breeding areas (off the coasts of Coquimbo and Concepción), where the contribution of the most representative fatty acids in both areas is evaluated. (DOCX)

S5 Table Analysis of the percentage of similarity (SIMPER) in fatty acids of the viscera and muscle of juvenile *Pleuroncodes monodon* females from two breeding areas, where the contribution of the most representative fatty acids is evaluated. (DOCX)

S1 File Environmental data of the sampling areas during an annual period (January to December of 2016) off the coast of Coquimbo and Concepción, Chile. (XLSX)

S2 File Female parameters data (i.e. cephalothorax length, total dry weight, lipid content, fatty acid profiles and fatty acid ratios) of visceral and muscle of juvenile *Pleuroncodes monodon* from two breeding areas (off the coasts of Coquimbo and Concepción). (XLSX)
