# Supplementary material for: Latitudinal changes in the lipid content and fatty acid profiles of juvenile female red squat lobsters (Pleuroncodes monodon) in breeding areas of the Humboldt Current System
Source: PLoS One. 2021 Jun 22;16(6):e0253314. doi: 10.1371/journal.pone.0253314 (PMC8219126; doi:10.1371/journal.pone.0253314)
Supplement: S1 Table — (DOCX) [file pone.0253314.s001.docx]

**S1 table.** **Generalized additive modelling of sea surface temperatures (SST) during an annual period (January to December of 2016) off the coast of Coquimbo and Concepción, Chile.**

| Parametric coefficients | Estimate | SE | t value | P value |
| --- | --- | --- | --- | --- |
| Intercept | 4.214 | 0.071 | 59.77 | < 0.0001 |
| Months (SST; NFU) | -0.037 | 0.009 | -3.841 | < 0.001 |
| Intercept | 15.31 | 0.339 | 45.13 | < 0.0001 |
| Months (SST; SFU) | -0.196 | 0.046 | -4.209 | < 0.001 |
