# Supplementary material for: Latitudinal changes in the lipid content and fatty acid profiles of juvenile female red squat lobsters (Pleuroncodes monodon) in breeding areas of the Humboldt Current System
Source: PLoS One. 2021 Jun 22;16(6):e0253314. doi: 10.1371/journal.pone.0253314 (PMC8219126; doi:10.1371/journal.pone.0253314)
Supplement: S2 Table — (DOCX) [file pone.0253314.s002.docx]

**S2 table. Generalized additive modelling of chlorophyll-a (Chl-a) during an annual period (January to December of 2016) off the coast of Coquimbo and Concepción, Chile.**

| Parametric coefficients | Estimate | SE | t value | P value |
| --- | --- | --- | --- | --- |
| Intercept | 0.624 | 0.244 | 2.554 | < 0.05 |
| Months (Chl-a; NFU) | 0.082 | 0.033 | 2.445 | < 0.05 |
| Intercept | 1.454 | 0.395 | 3.677 | < 0.001 |
| Months (Chl-a; SFU) | 0.036 | 0.054 | 0.672 | <0.05 |
