# Supplementary material for: Latitudinal changes in the lipid content and fatty acid profiles of juvenile female red squat lobsters (Pleuroncodes monodon) in breeding areas of the Humboldt Current System
Source: PLoS One. 2021 Jun 22;16(6):e0253314. doi: 10.1371/journal.pone.0253314 (PMC8219126; doi:10.1371/journal.pone.0253314)
Supplement: S3 Table — (DOCX) [file pone.0253314.s003.docx]

**S3 table**. **Statistics (one-way ANOVA for cephalothorax length, dry weight and lipid content, and two-way ANOVA for fatty acids and fatty acid ratios) of the differences in female parameters of viscera and muscle of juvenile *Pleuroncodes monodon* from two breeding areas (off the coasts of Coquimbo and Concepción).**

| Female parameters | Factor | df | MS | F | P |
| --- | --- | --- | --- | --- | --- |
| Cephalothorax length (mm) | Locality | 1 | 43.03 | 14.46 | < 0.001 |
|  | Error | 58 | 2.98 |  |  |
| Dry weight (mg) | Locality | 1 | 61203 | 3.69 | 0.059 |
|  | Error | 58 | 16585 |  |  |
| Lipids (mg g DW^-1^) | Locality | 1 | 0.643 | 52.18 | < 0.0001 |
|  | Organs | 1 | 1.354 | 109.87 | < 0.0001 |
|  | Locality x Organs | 1 | 0.003 | 0.25 | 0.778 |
|  | Error | 116 | 0.012 |  |  |
| Lipids (%) | Locality | 1 | 0.967 | 36.74 | < 0.0001 |
|  | Organs | 1 | 1.961 | 74.5 | < 0.0001 |
|  | Locality x Organs | 1 | 0.001 | 0.037 | 0.847 |
|  | Error | 116 | 0.026 |  |  |
| C16:0/C18:0 | Locality | 1 | 1.655 | 21.052 | < 0.0001 |
|  | Organs | 1 | 0.011 | 0.142 | 0.707 |
|  | Locality x Organs | 1 | 0.008 | 0.098 | 0.755 |
|  | Error | 92 | 0.079 |  |  |
| DHA/EPA | Locality | 1 | 0.393 | 1.407 | 0.239 |
|  | Organs | 1 | 5.774 | 2.066 | < 0.0001 |
|  | Locality x Organs | 1 | 0.251 | 0.899 | 0.346 |
|  | Error | 80 | 0.279 |  |  |
| PUFA/SFA | Locality | 1 | 0.148 | 0.352 | 0.554 |
|  | Organs | 1 | 0.006 | 0.015 | 0.902 |
|  | Locality x Organs | 1 | 0.012 | 0.028 | 0.868 |
|  | Error | 84 | 0.421 |  |  |
