# Supplementary material for: Latitudinal changes in the lipid content and fatty acid profiles of juvenile female red squat lobsters (Pleuroncodes monodon) in breeding areas of the Humboldt Current System
Source: PLoS One. 2021 Jun 22;16(6):e0253314. doi: 10.1371/journal.pone.0253314 (PMC8219126; doi:10.1371/journal.pone.0253314)
Supplement: S4 Table — (DOCX) [file pone.0253314.s004.docx]

**S4 table**. **Analysis of the percentage of similarity (SIMPER) in fatty acids of juvenile *Pleuroncodes monodon* females from two breeding areas (off the coasts of Coquimbo and Concepción), where the contribution of the most representative fatty acids in both areas is evaluated**.

| **Factor** | **Average similarity (%)** | **FA** | **Av.Abund.** | **Av.Sim.** | **Sim/SD** | **Contrib.%** | **Cum.%** |
| --- | --- | --- | --- | --- | --- | --- | --- |
| NFU | 57.84 | Palmitic (C16:0) | 4.09 | 17.52 | 2.11 | 30.29 | 30.29 |
|  |  | Oleic (C18:1ω9) | 2.34 | 8.99 | 2.08 | 15.54 | 45.83 |
|  |  | DHA (C22:6ω3) | 2.32 | 7.94 | 1.51 | 13.74 | 59.56 |
|  |  | Stearic (C18:0) | 1.60 | 7.74 | 2.00 | 13.38 | 72.94 |
|  |  | EPA (C20:5ω3)  Eicosatrienoic (C20:3ω3) | 1.68  1.03 | 6.14  1.68 | 1.49  0.44 | 10.62  2.90 | 83.56  86.46 |
|  |  | Palmitoleic (C16:1ω7) | 0.50 | 1.53 | 0.85 | 2.65 | 89.10 |
| SFU | 63.08 | Palmitic (C16:0) | 7.91 | 17.84 | 2.46 | 28.28 | 28.28 |
|  |  | Oleic (C18:1ω9) | 5.98 | 15.48 | 2.22 | 24.54 | 52.83 |
|  |  | DHA (C22:6ω3) | 3.97 | 7.92 | 1.67 | 12.55 | 65.38 |
|  |  | EPA (C20:5ω3) | 3.10 | 7.26 | 1.60 | 11.51 | 76.89 |
|  |  | Stearic (C18:0)  Palmitoleic (C16:1ω7) | 2.10  2.02 | 3.67  2.41 | 1.22  0.88 | 5.82  3.83 | 82.71  86.54 |

NFU, northern fishery unit; SFU, southern fishery unit; EPA, eicosapentaenoic acid; DHA, docosahexaenoic acid
