# Supplementary material for: Latitudinal changes in the lipid content and fatty acid profiles of juvenile female red squat lobsters (Pleuroncodes monodon) in breeding areas of the Humboldt Current System
Source: PLoS One. 2021 Jun 22;16(6):e0253314. doi: 10.1371/journal.pone.0253314 (PMC8219126; doi:10.1371/journal.pone.0253314)
Supplement: S5 Table — (DOCX) [file pone.0253314.s005.docx]

**S5 table**. **Analysis of the percentage of similarity (SIMPER) in fatty acids of the viscera and muscle of juvenile *Pleuroncodes monodon* females from two breeding areas, where the contribution of the most representative fatty acids is evaluated**.

| **Factor** | **Average similarity (%)** | **FA** | **Av.Abund.** | **Av.Sim.** | **Sim/SD** | **Contrib.%** | **Cum.%** |
| --- | --- | --- | --- | --- | --- | --- | --- |
| Viscera | 63.08 | Palmitic (C16:0) | 8.97 | 15.99 | 2.21 | 26.47 | 26.47 |
|  |  | Oleic (C18:1ω9) | 6.11 | 10.06 | 2.16 | 16.67 | 43.14 |
|  |  | DHA (C22:6ω3) | 4.64 | 8.27 | 1.80 | 13.69 | 56.83 |
|  |  | EPA (C20:5ω3) | 3.17 | 5.83 | 2.22 | 9.66 | 66.48 |
|  |  | Stearic (C18:0)  Myristic (C14:0)  Palmitoleic (C16:1ω7) | 2.63  1.71  2.16 | 5.72  2.66  2.65 | 1.98  1.50  1.09 | 9.47  4.41  4.38 | 75.95  80.36  84.74 |
| Muscle | 57.84 | Palmitic (C16:0) | 3.02 | 19.37 | 2.43 | 32.01 | 32.01 |
|  |  | Oleic (C18:1ω9) | 2.21 | 14.40 | 1.90 | 23.80 | 55.80 |
|  |  | DHA (C22:6ω3) | 1.65 | 7.60 | 1.41 | 12.55 | 68.36 |
|  |  | EPA (C20:5ω3) | 1.61 | 7.57 | 1.39 | 12.51 | 80.86 |
|  |  | Stearic (C18:0) | 1.07 | 5.69 | 1.16 | 9.40 | 90.26 |

EPA, eicosapentaenoic acid; DHA, docosahexaenoic acid.
